# Recurrent Adenomatous Neuroendocrine Tumor of the Middle Ear: A Diagnostic Challenge

**DOI:** 10.1155/2018/8619434

**Published:** 2018-11-25

**Authors:** J. Vilain, J.-C. Degols, S. Ledeghen

**Affiliations:** ENT Department, Clinique Saint-Pierre, 9 Av Reine Fabiola, 1340 Ottignies LLN, Belgium

## Abstract

Middle ear adenomatous neuroendocrine tumor (MEANT) poses a diagnostic challenge. Clinical symptoms are nonspecific. Definite diagnosis is made by histopathological analysis of the tumor after a complete surgical resection based on an extensive computed tomography/magnetic resonance imaging (CT/MRI). Controversial terminology of the neoplasm arises from the differentiation of this tumor composed of both endocrine and exocrine glands. Middle ear (ME) localization is rare and less aggressive than gastrointestinal tract or lung localizations. Nevertheless, clinical and CT/MRI analyses are necessary follow-ups for preventing or detecting recurrence or metastasis. A case of a female patient aged 26 with recurrent middle ear neuroendocrine adenoma is presented herein.

## 1. Introduction

Known for about 40 years [1, 2], MEANT still challenges pathologists and otorhinolaryngologists. ME localization is rare in comparison with gastrointestinal tract and lungs whose incidence is 2–5/100,000 [3]. Since the first case described in 1976 by Hyams and Michaels, about 150 cases have been reported in the English literature [4].

Clinical symptoms are nonspecific and include hearing loss, aural fullness, tinnitus, otalgia, facial weakness, and the like [5–9].

Anatomical imaging CT and MRI are not specific enough and sufficient for an accurate localization before surgery. Functional imaging (octreoscan™) targets somatostatin receptors, especially SSR2 and SSR5 located on the cell membrane of the tumor, and is much more sensitive [10, 11]. To date, this method is reserved for suspected cases with silent CT/MRI [4].

Definite diagnosis relies on combined histopathology and immunohistochemistry. The authors discuss the controversial terminology of these tumors based on different degrees of glandular or neuroendocrine differentiation [6, 7, 12, 13].

## 2. Case Report

In February 2008, a 26-year-old female patient consulted us for a second opinion before surgery on a suspected ME cholesteatoma. She had been complaining for several months about right aural fullness and otalgia.

**Figure 1:**
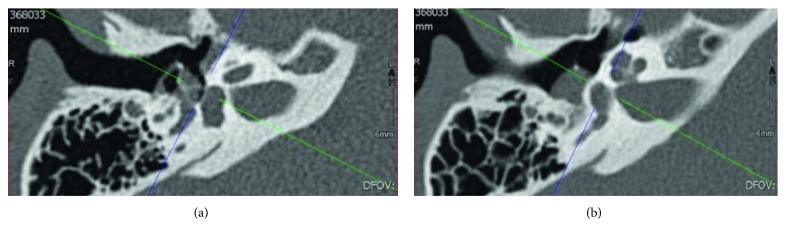
2008 high-resolution multidetector computed tomography (MDCT) (axial views) showing nonosteolytic nodular tissue mass in the hypotympanum alongside the promontory.

The otoscopy revealed a posterosuperior reddish retrotympanic mass without retraction pockets accompanied by a subnormal audiometry. The well-defined soft tissue mass density observed on the CT scan close to the ossicles but without any bone erosion did not support a diagnosis of cholesteatoma (cf.  Figure 1). A surgical exploration was performed, and the tumor easily resected through an ossicle preservation transmastoïd tympanoplasty.

**Figure 2:**
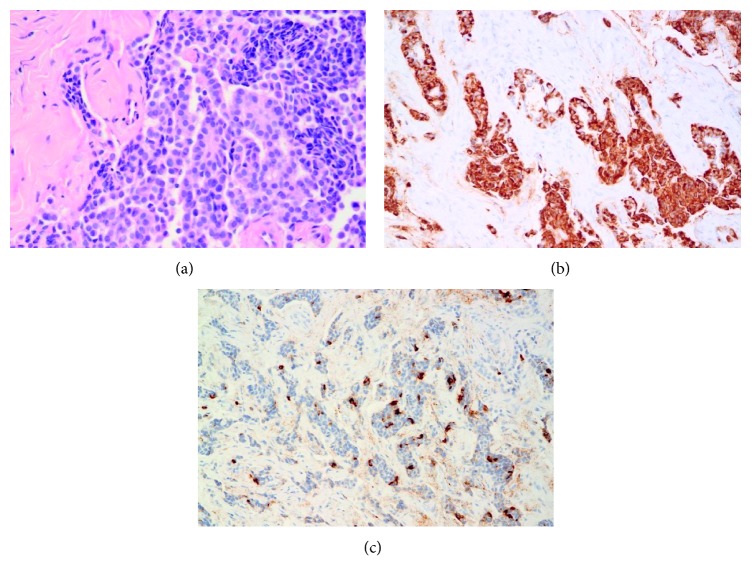
(a) Hematoxylin-eosin (HE) stain: the pattern of growth is trabecular or glandular. The tumor cells are uniform and cuboidal with a moderately abundant acidophilic cytoplasm. Mitoses are rare and necrosis absent. (b) Immunostaining for synaptophysin. (c) Immunoreactivity: typical immunostaining for chromogranin.

Histological examination revealed an epithelial proliferation with architecture sometimes trabecular, sometimes glandular, embedded in a dense fibrous stroma. The tumor was composed of uniform cuboidal or cylindrical cells with round to oval nuclei and a plasmacytoid morphology. No necrosis or mitotic activity was identified (cf.  Figure 2(a)). Periodic acid Schiff (PAS) staining revealed the presence of mucin in some cytoplasms.

Immunohistochemical staining was strongly positive for synaptophysin (cf.  Figure 2(b)), focally positive for chromogranin (cf.  Figure 2(c)) and neuron-specific enolase (NSE), reactive for epithelial membrane antigen (EMA) and cytokeratin (AE1/AE3) but was negative for S100 protein. The Ki67 cells proliferation index of 2% was weak. The tumor had the histological and immunohistochemical profile of a “carcinoid tumor.”

**Figure 3:**
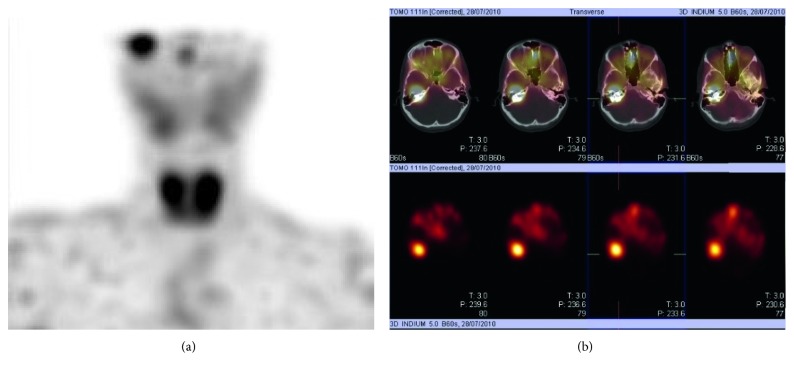
(a) ^111^In-pentetreotide scintigraphy from 2010 (MIP) intense activity in the right ME. (b) ^111^In-pentetreotide scintigraphy from 2010 (fused transverse SPECT/CT images and transverse SPECT images).

**Figure 4:**
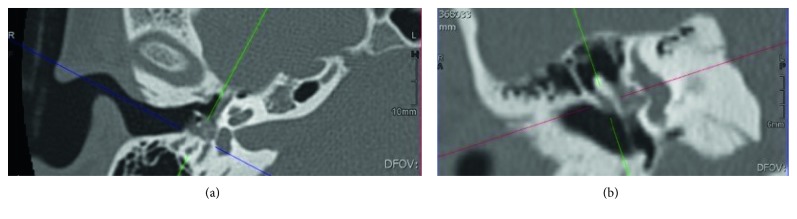
High-resolution MDCT from 2010 (coronal and axial views): nodular tissue mass located in the mesotympanum covering the incus and the stapes without osteolysis, suspected of tumor recurrence.

Two years after the tumor resection, the patient presented an intense right otalgia and a neurosensorial hearing loss. ^111^Indium-pentetreotide scintigraphy showed an intense activity in the right ME (cf.  Figure 3), and the CT scan revealed a soft tissue density mass in the attic of the ME, which confirmed the MEANT recurrence (cf.  Figure 4). During a revision surgery, a yellowish tissue was resected by removing the ossicular chain. No adherence or bone erosion was noted. Function was restored by a total ossicular replacement prosthesis (TORP) ossiculoplasty. Histopathology disclosed the neuroendocrine (NE) nature of the tumor.

Scintigraphy and CT scan were free of recurrence two years after the revision surgery. The patient came for her follow-up check 10 years later without any complaint, and follow-up CT scan was negative.

## 3. Discussion

Histology and immunohistochemistry are the keys for definite diagnosis of the MEANT. These analyses describe classically both exocrine (mucinous) and/or neuroendocrine differentiation. Immunoreactivity is variously positive for keratin antibodies (cytokeratin, CK7, CK20, and CAM5.2) and neuroendocrine markers (chromogranin, synaptophysin, serotonin, nonspecific enolase, and human pancreatic polypeptide) [6, 12]. Diversity of expression and clinical behavior of these tumors explain the variant terminology used to describe them, including middle ear adenoma, adenomatous tumor, neuroendocrine adenoma, carcinoid tumor, and mixed epithelial and neuroendocrine tumor [13, 14].

Coexistence of cells derived embryologically from both endoderm and mesoderm (neural crest) led some authors to claim either that uncommitted epithelial (endodermal) cells would develop neuroendocrine features, or that uncommitted neural crest cells would acquire exocrine and epithelial characteristics [6].

Most cases of ME localization in literature are described in case reports with local malignancy. Recurrence rate of insufficiently resected tumors occurs between 15 and 22% according to the authors [9, 15]. Even if some authors report cervical nodes and osseous metastases [10, 16], MEANT is less aggressive than gastrointestinal tract or lung localization, as there is absence of necrosis and a low mitotic rate by histologic analysis.

The best treatment is surgery, and the extent of resection depends on the staging, from intact canal wall tympanomastoïdectomy to a subtotal petrosectomy.

Saliba and Evrard [5] reviewed 75 cases and proposed a classification of ME glandular neoplasm based on NE markers immunoreactivity and presence or not of metastases. The average follow-up of the 73 disease-free patients after definitive surgery was 53 months, whereas the average time of recurrence or metastases was 11 years. NE neoplasm behavior remains unpredictable.

More recently, Marinelli et al. [4] proposed through a multi-institutional retrospective study on 32 cases a T/N/M/S staging system for MEANTs. About a third of T2 MEANTs and nearly two-thirds of T3 MEANTs developed local recurrence at a median duration of recurrence of 6 years. According to this staging, our patient is classified as T2a.

MEANTs are listed in the 2017 WHO classification as “middle ear adenoma,” arguing that it is always appropriate to designate them as adenomas with neuroendocrine features [17].

Shaverdian et al. [18] studied the clinical relevance of ^111^In-pentetreotide scintigraphy in the diagnosis and the management of 145 patients with NE tumors (particularly gastrointestinal tract and lung localization). The authors noted that cross-sectional CT/MRI detected 60% of primary or recurrent tumors and suggested that scintigraphy should be reserved for patients with biochemical diagnosis of NE tumor with silent CT/MRI.

## 4. Conclusions

MEANT is a rare, slow-growing, and low-grade malignancy tumor with nonspecific clinical symptoms. Radical surgery treatment prevents 22% recurrence. Clinical combined with CT/MRI remains actually the best available method for controlling recurrence. Octreotide scintigraphy will be performed in case of doubt. NE neoplasm behavior, however, still remains unpredictable, and a lifelong follow-up is necessary.
